# *Candida albicans* adhesion, biofilm formation and gene expression on conventional, milled and 3D-printed denture base materials: an in vitro study

**DOI:** 10.1186/s12903-026-10345-y

**Published:** 2026-11-04

**Authors:** Julia Roßbach, Christoph Sasse, Ralf Bürgers, Oskar Bunz

**Affiliations:** Department of Prosthodontics, University Medical Center Göttingen, Robert-Koch-str 40, Göttingen, 37075 Germany

**Keywords:** Denture Bases, *Candida albicans*, Biofilm, Surface Properties, CAD/CAM, 3D Printing, Prosthesis-Related Infections, Fungal Gene Expression Regulation

## Abstract

**Background:**

Denture base materials are colonized by *Candida albicans*, and surface properties influence microbial adhesion. Evidence on subtractively and additively manufactured materials remains limited. This in vitro study examined surface characteristics of conventional, milled and 3D-printed denture base materials and their association with *C. albicans* adhesion, biofilm formation and gene expression.

**Methods:**

Specimens of eight conventional, milled and 3D-printed denture base materials were polished to an arithmetic surface roughness (R_a_) below 0.2 μm and characterized by surface free energy (SFE), scanning electron microscopy and energy-dispersive X-ray spectroscopy. Initial adhesion of *C. albicans* DSM 1386 (2 h) was quantified by fluorescence staining. For the materials with the highest and lowest adhesion (Temp Basic, Denture Gingiva Basic Mono), 24-h biofilm formation was quantified by crystal violet staining, and *ALS3* and *EFG1* expression by RT-qPCR after 2, 4 and 8 h, additionally in two oral clinical isolates. Data were analyzed using (Welch) ANOVA with post hoc tests and one-sample t-tests (α = 0.05).

**Results:**

Temp Basic showed a significantly lower SFE than all other materials (*p* ≤ 0.012) and the highest mean initial adhesion, which differed significantly from PalaXpress, V-Print dentbase and Denture Gingiva Basic Mono (*p* ≤ 0.018). Biofilm biomass after 24 h did not differ between Temp Basic and Denture Gingiva Basic Mono (*p* = 0.261). In the reference strain, *ALS3* and *EFG1* expression was significantly higher on Denture Gingiva Basic Mono after 4 h (p_*ALS3*_ = 0.009; p_*EFG1*_ = 0.038), but not after 28 h. Both clinical isolates likewise showed higher expression after 4 h, without significance in clinical isolate 1; clinical isolate 2 was tested in a single experiment.

**Conclusions:**

Within the limitations of this in vitro study, SFE alone did not explain material-dependent differences in initial *C. albicans* adhesion, and higher initial adhesion was not associated with greater biofilm biomass. *ALS3* and *EFG1* expression after 4 h was higher on Denture Gingiva Basic Mono, significantly in the reference strain and in the same direction in two clinical isolates. Confirmation under clinically relevant conditions, including a salivary pellicle and multispecies biofilms, is required.

**Supplementary Information:**

The online version contains supplementary material available at https://doi.org/10.1186/s12903-026-10345-y.

## Background

Opportunistic infections in elderly and immunocompromised individuals represent a growing clinical concern. A local predisposing factor for an increased prevalence of opportunistic oral infections like denture stomatitis is the utilization of removable dentures [1]. Denture stomatitis can adversely affect general health and is primarily associated with colonization of the denture base surface by *Candida albicans* (*C. albicans*), for which the material provides a substratum for attachment and subsequent colonization [2–6].

Biofilm development proceeds through a series of distinct stages. It commences with the adhesion of microbial cells, which is then followed by proliferation and the formation of a mature biofilm. Within this environment, intermicrobial communication can occur [7–9]. This process is governed by the physicochemical properties of the material surface, which can lead to differences in the microbial adhesion process. Such properties include surface roughness, surface free energy (SFE), wettability and the chemical composition of the surface [10–13]. Surface roughness provides retention sites that protect microorganisms from shear forces; below a threshold R_a_ of 0.2 μm, originally determined for titanium abutments, its influence on microbial adhesion is considered negligible [14]. SFE and wettability determine the thermodynamics of adhesion through hydrophobic interactions [10, 11], whereas the elemental surface composition, e.g. inorganic fillers or pigments, may alter surface charge and thereby affect electrostatic and acid–base interactions [13, 15]. Energy-dispersive X-ray spectroscopy (EDS) enables a qualitative assessment of such elemental surface constituents. Because these differ between denture base materials, so does their influence on adhesion [13, 16, 17]. Beyond adhesion, interactions between the material and microorganisms can trigger biological reactions like gene expression that go beyond adhesion [10, 11].

*C. albicans*, a commensal and opportunistic pathogenic fungus of the oral microbiota, is present in the oral cavity and participates in biofilm formation on denture base materials [2, 18, 19]. Its behavior is governed by a complex network of virulence-associated genes that influence adhesion, morphogenesis, and biofilm formation. The fungus manifests in two distinct morphological forms, the blastospore and the hyphal form [20]. The hyphal form is associated with epithelial invasion and damage, mediated in part by the hyphae-associated peptide toxin candidalysin, and shows greater inflammatory and virulence potential [18, 21–23]. The transition to the hyphal form is driven by the regulated expression of specific genes [19–21]. Within this regulatory network, the transcription factor *EFG1*, together with other factors, assumes a pivotal role in the processes of morphogenetic switching and biofilm formation [24, 25], whereas *ALS3*, which encodes a hypha-associated adhesin, is regarded as a key determinant of surface adhesion and biofilm formation [26].

Microbial gene expression can also be modulated by polymer materials [27]. For example, in denture base materials containing piezoelectric particles such as barium titanate, both the particles and cyclic mechanical stress significantly altered the expression of virulence-associated genes [28]. Comparable effects have been reported for bacteria. In *Staphylococcus epidermidis* and methicillin-resistant *Staphylococcus aureus*, the incorporation of hydroxypropyltrimethyl ammonium chloride chitosan into polymethyl methacrylate (PMMA) bone cement reduced the expression of virulence-associated genes such as *icaAD* [29]. However, direct evidence for such effects in a dental context remains limited, and further studies are required.

Besides conventional heat- or autopolymerizing PMMA processed by compression molding or injection techniques, digital workflows have become established for denture base fabrication. In subtractive manufacturing, the denture base is milled from industrially prepolymerized PMMA blanks, which are polymerized under high pressure and temperature and are therefore characterized by low porosity and low residual monomer content. In additive manufacturing, the denture base is built layer by layer from light-curing (meth)acrylate-based resins, usually by stereolithography or digital light processing, followed by post-curing [30]. As composition, degree of conversion and processing vary between these techniques, surface properties and interactions with microorganisms may vary as well [31].

The physicochemical properties of conventionally and newly manufactured denture base materials, and their influence on microbial adhesion, have been investigated extensively [16, 17, 31–33].

Several studies have compared *C. albicans* adhesion and biofilm formation on conventional, milled and 3D-printed denture base materials, with partly contradictory results [31, 34–36]. Material-dependent effects on *C. albicans* gene expression, however, have mainly been investigated for experimentally modified or mechanically loaded PMMA [27–29], whereas commercially available denture base materials from conventional, subtractive and additive manufacturing have not yet been compared in this respect. We therefore hypothesized that denture base materials from conventional, subtractive and additive manufacturing vary in their surface characteristics and that these characteristics are associated with the number of initially adhering *C. albicans* cells, the expression of biofilm-associated genes and the amount of biofilm formed.

Accordingly, the aims of this study were (i) to characterize eight denture base materials with respect to surface roughness, SFE, surface morphology (SEM) and elemental surface composition (EDS); (ii) to quantify the initial adhesion of *C. albicans* to these materials; and, for the two materials with the highest and lowest initial adhesion, (iii) to quantify biofilm formation after 24 h and (iv) to analyze the expression of the adhesin gene *ALS3* and the transcription factor gene *EFG1* during early biofilm development in a reference strain and two clinical isolates.

## Methods

### Fabrication of test specimens

Cylindrical specimens (diameter 10 mm) were fabricated from eight denture base materials according to the manufacturers’ instructions; their final thickness depended on the manufacturing process (see below). The software and equipment used are listed in Table 1. Specimens were allocated to the experiments arbitrarily, without a formal randomization procedure, and evaluations were not blinded.

**Table 1:** Denture base materials investigated and their manufacturing characteristics

Denture base material	Manufacturer	Manufacturing process	Software	Equipment
Paladon 65	Kulzer GmbH, Hanau, Germany	Conventional (injection molding)	Zirkonzahn.Software (Zirkonzahn SRL, Val Pusteria, South Tyrol, Italy)	M5 Heavy Metal Milling Unit (Zirkonzahn SRL, Val Pusteria, South Tyrol, Italy)
PalaXpress	Kulzer GmbH, Hanau, Germany	Conventional (injection molding)		
V-Print dentbase	VOCO GmbH, Cuxhaven, Germany	Additive (3D printing)	W2P (W2P Engineering GmbH, Wien, Austria)	SolFlex 350 and Otoflash (VOCO GmbH, Cuxhaven, Germany) Bandelin Sonorex RK 100 (BANDELIN, Berlin, Germany)
Denture Base Resin (color: OP)	Formlabs Inc., Somerville, USA	Additive (3D printing)	PreForm (Formlabs Inc., Somerville, USA)	Form 3B+, Form Wash and Form Cure (Formlabs Inc., Somerville, USA)
Temp Basic (color: Tissue A)	Zirkonzahn GmbH, Pustertal, Italy	Subtractive (milling)	Zirkonzahn.Software (Zirkonzahn SRL, Val Pusteria, South Tyrol, Italy)	M5 Heavy Metal Milling Unit (Zirkonzahn SRL, Val Pusteria, South Tyrol, Italy)
Denture Gingiva Basic Mono (color: pink)	Zirkonzahn GmbH, Pustertal, Italy	Subtractive (milling)		
M-PM Disc (color: pink)	Merz Dental GmbH, Lütjenburg, Germany	Subtractive (milling)	Remote DENTAL (imes-icore GmbH, Eiterfeld, Germany)	imes-icore CORiTEC 350i series (imes-icore GmbH, Eiterfeld, Germany)
Ivotion Base (color: Pink-V)	Ivoclar Vivadent GmbH, Ellwangen, Germany	Subtractive (milling)	PrograMill CAM (Ivoclar Vivadent Schweiz AG, Glattpark [Opfikon], Switzerland)	PrograMill PM7 (Ivoclar Vivadent Schweiz AG, Glattpark [Opfikon], Switzerland)

For the conventionally manufactured materials, the resin was injected into a mold with the dimensions of a milling disc. From these discs and from the milling discs of the subtractively manufactured materials, six cylinders (diameter 10 mm) each were milled and sliced into discs using a precision cutting machine (Micracut 201, Metkon, Bursa, Turkey).

The additively manufactured specimens were printed directly in cylindrical form. V-Print dentbase was printed with a build orientation of 90° and a layer thickness of 50 μm, cleaned in 2-propanol (≥ 99.5%, Carl Roth GmbH + Co. KG, Karlsruhe, Germany) in an ultrasonic bath (Bandelin Sonorex RK 100) in two steps (3 min pre-cleaning, 2 min final cleaning) and post-cured in the Otoflash unit (2 × 2000 flashes). Denture Base Resin was printed with a build orientation of 0° and a layer thickness of 50 μm, and washed and post-cured according to the manufacturer’s instructions.

All specimens were then polished with an automated grinding machine (Digiprep 251, Metkon, Bursa, Turkey) to an arithmetic surface roughness (R_a_) below 0.2 μm by utilizing material-specific polishing protocols (Table S1) and the following grinding papers: DEMA-PL grinding paper discs adhesive (Metkon Instruments Inc., Bursa, Turkey), WS Flex 18 C SK grit 400 and 1200, as well as WS Flex 18 CMS grit 4000 (Hermes Schleifmittel GmbH, Hamburg, Germany). After polishing, the final specimen thickness was 1.7–2.0 mm for the conventionally, 2.2–2.5 mm for the subtractively and 1.2–1.6 mm for the additively manufactured materials.

### Surface roughness

The R_a_ was measured at 20× magnification using a computer-controlled confocal wide-field microscope (ZEISS Smartproof 5, Carl Zeiss IQS Germany GmbH, Oberkochen, Germany) and the corresponding software. The measurement was conducted at five distinct points per test specimen, with a total of five test specimens per denture base material.

### Surface free energy

SFE was measured on the polished side of the specimen using the sessile drop method with a contact angle measuring device (Drop Shape Analyzer DSA25, KRÜSS GmbH Wissenschaftliche Laborgeräte, Hamburg, Germany) on five specimens per denture base material. Ten separate measurements were taken of each specimen at 1 s intervals using diiodomethane (Sigma-Aldrich Chemie GmbH, Taufkirchen, Germany) and double-distilled water. The SFE was calculated using an analyzing software (ADVANCE, KRÜSS GmbH Wissenschaftliche Laborgeräte, Hamburg, Germany) based on the static contact angles determined using the Owens-Wendt method.

### Scanning Electron Microscopy (SEM) and Energy-Dispersive X-ray Spectroscopy (EDS)

The specimens for SEM imaging and EDS were prepared as described below for the initial adhesion assay. An ascending alcohol series ranging from 70% to ≥ 99.8% ethanol (Carl Roth GmbH + Co. KG, Karlsruhe, Germany) followed for dehydration. The surface was sputtered with a 60% gold and 40% palladium alloy until a 20–30 nm layer thickness was achieved (Cressington 208HRD turbo sputter coater, Micro to Nano, Haarlem, Netherlands). SEM imaging and EDS analysis were performed using a scanning electron microscope (JEOL JSM-IT510 InTouchScope™, JEOL GmbH, Freising, Germany). The representative SEM images of the polished surfaces were acquired from the same areas selected for EDS analysis. The EDS analysis was conducted on a single test specimen for each type of denture base material with five randomly selected measuring points per specimen. As EDS was performed on one specimen per material, it served exclusively for the qualitative characterization of the elemental surface composition. The voltage was set to 15 kV and the EDS analysis was performed by analyzing the K-lines of the elements.

### C. albicans strains and culture conditions

*C. albicans* DSM 1386 (Deutsche Sammlung von Mikroorganismen und Zellkulturen, DSMZ, Braunschweig, Germany), originally isolated in 1978 from a patient with bronchomycosis, was cultured under standard conditions in Universal Medium for Yeast (YM; 10 g/l glucose, 3 g/l yeast extract, 3 g/l malt extract, 5 g/l peptone, pH = 6.5). The culture medium was prepared according to the manufacturer’s instructions using 1 l double-distilled water, 3 g yeast extract (Sigma-Aldrich Chemie GmbH, Taufkirchen, Germany), 3 g malt extract (Sigma-Aldrich Chemie GmbH, Taufkirchen, Germany), 5 g peptone from casein (Merck KGaA, Darmstadt, Germany) and 10 g D(+)-glucose monohydrate (Merck KGaA, Darmstadt, Germany) adjusted to a pH of 6.5.

### Clinical isolates

Two clinical *C. albicans* isolates from the oral cavity, referred to as clinical isolate 1 (internal designation Peu 3962) and clinical isolate 2 (internal designation Peu 3309), were provided by the Department of Medical Microbiology and Virology, University Medical Center Göttingen. They were streaked onto yeast extract–peptone–dextrose (YPD) agar plates and incubated overnight at room temperature. The YPD medium was prepared with 1 l double-distilled water, 10 g yeast extract (Sigma-Aldrich Chemie GmbH, Taufkirchen, Germany), 20 g peptone from casein (Merck KGaA, Darmstadt, Germany), 20 g D(+)-glucose monohydrate (Merck KGaA, Darmstadt, Germany) and 20 g agar (Agar-Agar Kobe I, Carl Roth GmbH + Co. KG, Karlsruhe, Germany), and poured into petri dishes with ventilation cams (SARSTEDT AG & CO. KG, Nümbrecht, Germany).

On the following day, colonies were transferred with an inoculation loop into 6 ml YM and cultured overnight under standard conditions.

### Initial adhesion (fluorescence staining)

Specimens were cleaned with 70% ethanol (Th Geyer GmbH & Co. KG, Renningen, Germany) for 5 min and then glued in place 24 h before the start of the experiment using duplicating silicone (Z-Dupe duplicating silicone, Henry Schein Dental Germany GmbH, Langen, Germany) and an inoculation loop. After a drying time of 10 min, as required by the manufacturer the materials PalaXpress and Paladon 65 were coated with 1000 µl of double-distilled water for 12 h.

Microorganisms were centrifuged at 2268 × *g* by centrifuge (Thermo Scientific™ Megafuge 8R, Thermo Fisher Scientific, Waltham, Massachusetts, USA) and washed twice via 30 ml phosphate buffered saline (PBS) (Merck, Darmstadt, Germany). After resuspension with 10 ml PBS the optical density was adjusted to 0.1 at 600 nm using a photometer (BioPhotometer, Eppendorf SE, Hamburg, Germany) by adding PBS. Microbial adhesion was performed by adding 1000 µl of *C. albicans* to each well followed by an incubation for 2 h at 37 °C and 0.46 × *g*. After performing a washing step using 1 ml of 0.85% NaCl, 800 µl of the Hoechst 33342 dye solution was pipetted in a ratio of 5 µl Hoechst to 1 ml 0.85% NaCl and incubated again at 0.46 × *g* and 22.3 °C for 12 min. Four further washing steps were carried out as described above, with the third and fourth washing steps each followed by incubation for 5 min at 0.46 × *g*. The microorganisms were fixed by adding 800 µl of 2% paraformaldehyde solution (PFA) (Merck KGaA, Darmstadt, Germany) and then incubated for 15 min at 22.3 °C and 0.46 × *g*. After two further washing steps and light-protected drying of the test specimens for 1 h the specimens were placed on cover glasses (Th Geyer GmbH & CO. KG, Renningen, Germany). Using the fluorescence microscope (KEYENCE Germany GmbH, Neu-Isenburg, Germany) with blue filter DAPI OP-87762 an overview image was taken at 4× magnification and five images each at 40× magnification were taken, selecting the blue and yellow channels. The five fields were positioned approximately in a quincunx pattern. Adherent cells were counted in each image using Fiji ImageJ (version 2.9.0/1.53t; Rasband WS, US National Institutes of Health, Bethesda, Maryland, USA), and the mean of the five images was calculated per specimen. Images were converted to 16-bit, calibrated using the 50 μm scale bar and segmented with an individually adjusted threshold; touching cells were separated using the Watershed function and counted using Analyze Particles.

### Biofilm formation (crystal violet assay)

Biofilm formation was analyzed on Temp Basic and Denture Gingiva Basic Mono. Temp Basic and Denture Gingiva Basic Mono were selected because they showed the highest and lowest mean initial adhesion, respectively, and thus represented the extremes of initial colonization. The specimens were washed in 70% ethanol for 15 min in a beaker, followed by drying in a 24-well plate. To ascertain the quantity of biofilm, a total of seven biological replicates were conducted for each material, with each replicate comprising three technical replicates. Specimens incubated only with YM were used as negative control. For colonization, *C. albicans* cells were centrifuged at 2101 × *g* and washed twice with 30 ml PBS. After resuspension in 10 ml YM, the optical density was adjusted to 0.5 by adding YM. Microbial adhesion was performed by adding 1 ml of *C. albicans* suspension to each well followed by an incubation for 24 h at 37 °C and 0.794 × *g*.

After removing the supernatant, two washing steps using 1 ml of PBS were performed and the specimens were transferred to a new plate. 1 ml of 0.1% crystal violet (C.I.42555, Carl Roth GmbH + Co. KG, Karlsruhe, Germany) was pipetted onto each test specimen and incubated for 15 min at room temperature. After removing the crystal violet solution, two washing steps were performed using 1 ml PBS and the specimens were transferred to a new 24-well plate. This was followed by another wash cycle. 1 ml of 90% ethanol was added to each sample and incubated for 15 min. From each supernatant obtained, three 100 µl aliquots of the dye solution were taken and transferred to a 96-well plate (SARSTEDT AG & CO. KG, Nümbrecht, Germany). The OD_595_ was then measured using a microplate reader (FLUOstar Omega, BMG LABTECH, Ortenberg, Germany) and the associated software (Omega Control 5.11, BMG LABTECH, Ortenberg, Germany). The data sets were analyzed using MARS Data Analysis Software (BMG LABTECH, Ortenberg, Germany). For the analysis, the OD of the supernatants from the negative control specimens was subtracted from that of the colonized test specimens. To account for inter-experimental variability, values were normalized to Denture Gingiva Basic Mono within each independent experiment: the mean of Denture Gingiva Basic Mono was set to 1, and the values of Temp Basic were expressed relative to it. The normalized values were therefore compared with the hypothetical value of 1 (no difference) using a one-sample t-test.

### ALS3 and EFG1 expression (RT-qPCR)

The regulation of *ALS3* and *EFG1* of *C. albicans* was investigated using reverse transcription quantitative PCR (RT-qPCR) after incubation periods of 2 h, 4 h and 8 h in YM. *TDH3* was used as a reference [37]. For the reference strain and clinical isolate 1, three biological replicates, each with two technical replicates (duplicate RT-qPCR reactions from the same cDNA), were analyzed; clinical isolate 2 was analyzed in a single experiment. The test specimens were prepared and the OD of *C. albicans* was adjusted to 0.5. Then, the specimens were washed with PBS and dried at room temperature for 10 min. RNA was isolated using a RNA isolating kit (YeaStar RNA Kit R1002, Zymo Research Europe GmbH, Freiburg, Germany) according to the manufacturer’s protocol with one modification. After incubation with *C. albicans*, the specimens were placed in an ultrasonic cleaner (Sonorex Super RK 31 ultrasonic device, BANDELIN electronic GmbH & Co. KG, Berlin, Germany) for 5 min before adding zymolase. The RNA quantities in the samples were determined using a spectrophotometer (NanoDrop 1000, Peqlab Biotechnologie GmbH, Erlangen, Germany).

The subsequent DNase I digestion was performed according to the instructions for DNase I (Zymo Research Europe GmbH, Freiburg, Germany), using 1 µg of the respective RNA. To verify sufficient DNase I digestion, a control qPCR was performed using DNase-treated RNA as template with the *TDH3* gene as target over 35 cycles on a magnetic induction cycler (mic qPCR cycler, BMS Bio Molecular Systems, Upper Coomera, Australia).The renewed determination of the RNA concentration was followed by transcription of the RNA into cDNA using a cDNA synthesis kit (Biozym cDNA Synthesis Kit, Biozym Scientific GmbH, Hessisch Oldendorf, Germany) with the magnetic induction cycler (mic qPCR cycler thermocycler, bms Bio Molecular Systems, Upper Coomera, Australia). The kit was used according to the instructions.

The RT-qPCR was performed using a qPCR mastermix (Blue S’Green qPCR Mix Separate ROX kit, Biozym Scientific GmbH, Hessisch Oldendorf, Germany). The analysis was performed using an analyzing software (Mic qPCR Analysis Software, bms Bio Molecular Systems, Upper Coomera, Australia). Primer sequences are listed in Table 2. Relative expression was calculated using the ΔΔCq method: Cq values of *ALS3* and *EFG1* were normalized to *TDH3* (ΔCq), the ΔCq values obtained on Denture Gingiva Basic Mono were related to those obtained on Temp Basic (ΔΔCq), and fold changes (2^−ΔΔCq^) were calculated with Temp Basic set to 1.

**Table 2:** Primer sequences used for RT-qPCR

Gene	Forward primer (5′→3′)	Reverse primer (5′→3′)
*ALS3*	GGAGCATTACCACCACCATT	GCTTCATCGCCTATCATTTCTT
*EFG1*	TAATGTGTCTGGTGCTTCTGGT	CTGCTGTTGGTTGTAAGTTTGA
*TDH3*	CATTGAAAGGTGTTTTGGGCTA	ATTTGACGAAAGTTGGGGACA

### Statistical analysis

All data were analyzed using GraphPad Prism (version 9.4.1, GraphPad Software, Boston, MA, USA) with a significance level of α = 0.05. Normal distribution was assessed using the Shapiro-Wilk test (SFE) or the Anderson-Darling test (initial adhesion), and homogeneity of variances using Bartlett’s test. As variances of SFE were not homogeneous, SFE values were compared using Welch’s analysis of variance (ANOVA) followed by the Games-Howell post hoc test. Initial adhesion was compared using ordinary one-way ANOVA followed by Tukey’s multiple comparison test. Fold changes in gene expression (Temp Basic = 1) and normalized biofilm data (Denture Gingiva Basic Mono = 1) were compared with the hypothetical value of 1 using a one-sample t-test. Gene expression data of clinical isolate 2 (*n* = 1) were evaluated descriptively without statistical testing.

To explore a possible association between SFE and biofilm biomass, SFE was additionally measured on each specimen used for the crystal violet assay. As the data were not normally distributed, Spearman’s rank correlation was calculated for both materials combined and for each material separately, to exclude that an association was masked by material-specific differences. In addition, a multiple linear regression with biofilm biomass as dependent variable and SFE and material as independent variables was performed; owing to heteroscedastic residuals, biofilm values were log-transformed. Results were visualized as a scatter plot with separate regression lines for both materials (Fig. S1).

## Results

### Surface characterization

**Fig. 1:**
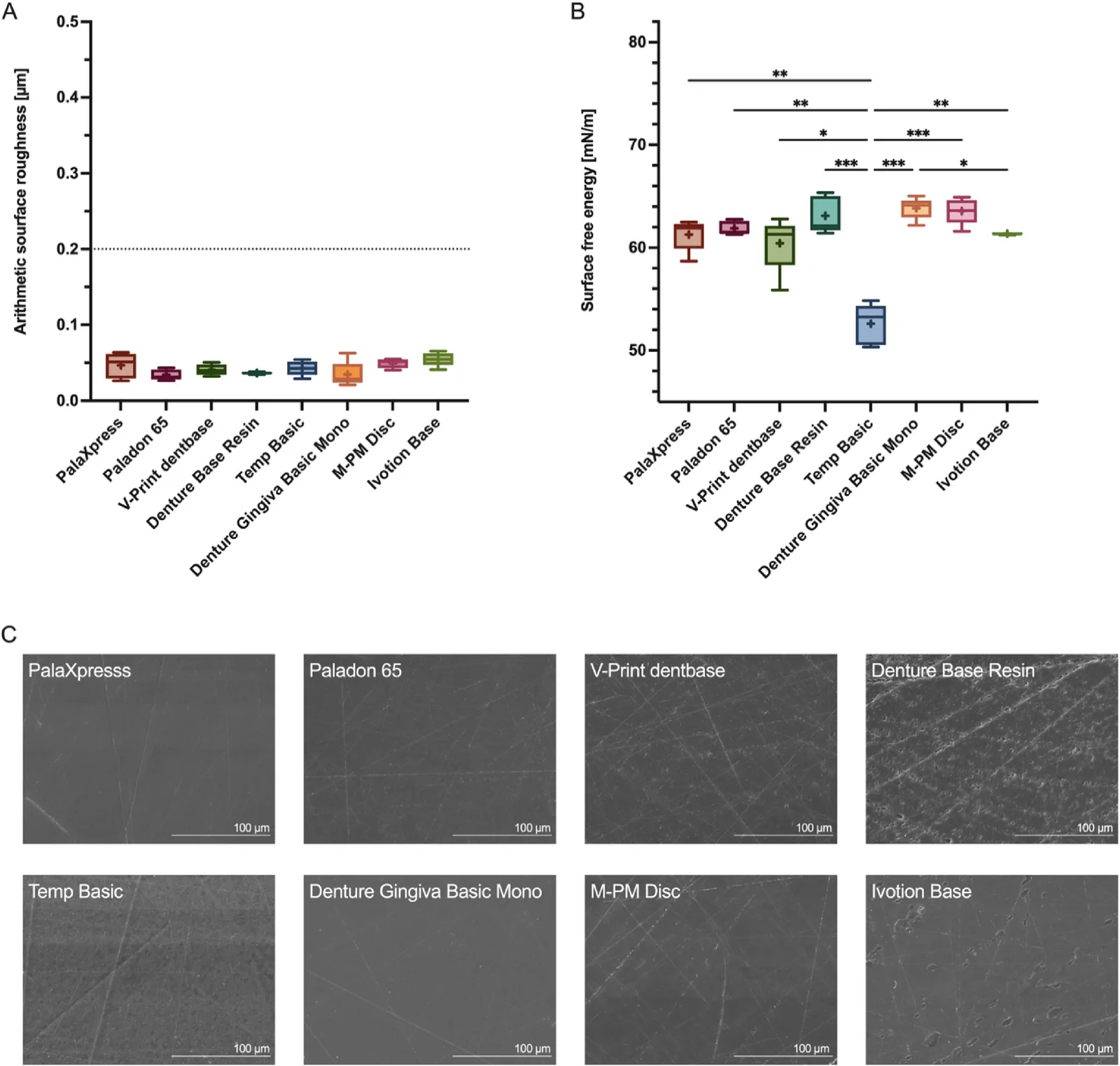
Surface characterization of the denture base materials. **A **Arithmetic surface roughness (R_a_) after material-specific polishing; dotted line: threshold of 0.2 μm. **B** Surface free energy (SFE). Statistical analysis was performed using Welch’s ANOVA with the Games-Howell post hoc test. Differences were considered statistically significant at *p* < 0.05. Asterisks (*) indicate * *p* < 0.05; ** *p* < 0.01; *** *p* < 0.001; *n* = 5; + = mean. **C** Representative SEM images of the polished surfaces; scale bar = 100 μm (lower right corner). Material names are indicated in the upper left corner

The R_a_ of all materials was below 0.2 μm (Fig. 1A; Table S3). Temp Basic showed a significantly lower SFE than all other materials (*p* ≤ 0.012), and SFE also differed significantly between Denture Gingiva Basic Mono and Ivotion Base (*p* = 0.045; Fig. 1B). SEM images showed polishing grooves on all materials, which appeared more pronounced on Denture Base Resin (Fig. 1C). EDS detected carbon (C) and oxygen (O) as the main elements on all materials. In addition, aluminum (Al) was detected on Paladon 65 and Temp Basic, Al and silicon (Si) on V-Print dentbase, and Al, potassium (K), titanium (Ti), iron (Fe), manganese (Mn) and phosphorus (P) on Denture Base Resin (Table S2).

### Initial adhesion

**Fig. 2:**
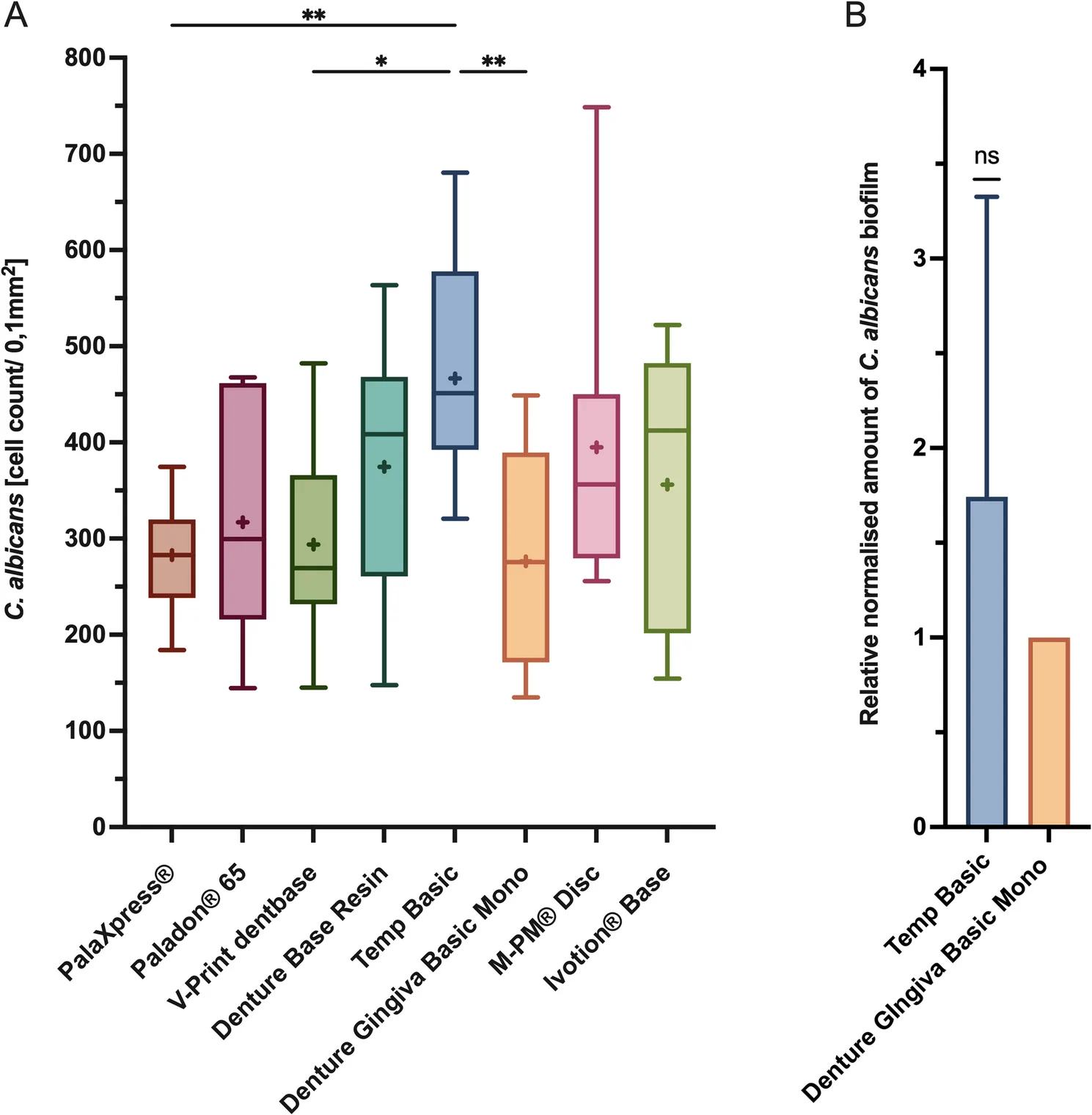
Initial adhesion (**A**) and biofilm formation after 24 h (**B**) of *C. albicans*. **A **Number of *C. albicans* cells adhering to the eight denture base materials after 2 h. Statistical analysis was performed using one-way ANOVA with Tukey’s multiple comparison test. Asterisks indicate * *p* < 0.05; ** *p* < 0.01; *n* = 11; + = mean. **B** Biofilm biomass after 24 h on Temp Basic and Denture Gingiva Basic Mono only (crystal violet assay). Statistical analysis was performed using a one-sample t-test against the hypothetical value of 1. ns = not significant; *n* = 7

After 2 h, the mean number of adherent *C. albicans* cells ranged from 276.4 (Denture Gingiva Basic Mono) to 466.5 cells/0.1 mm² (Temp Basic) (Fig. 2A; Table S3). Initial adhesion on Temp Basic was significantly higher than on PalaXpress (*p* = 0.009), V-Print dentbase (*p* = 0.018) and Denture Gingiva Basic Mono (*p* = 0.006); no other pairwise comparison was statistically significant.

### Biofilm formation

After 24 h, biofilm biomass did not differ significantly between Temp Basic and Denture Gingiva Basic Mono (*p* = 0.261; Fig. 2B).

### Expression of ALS3 and EFG1

**Fig. 3:**
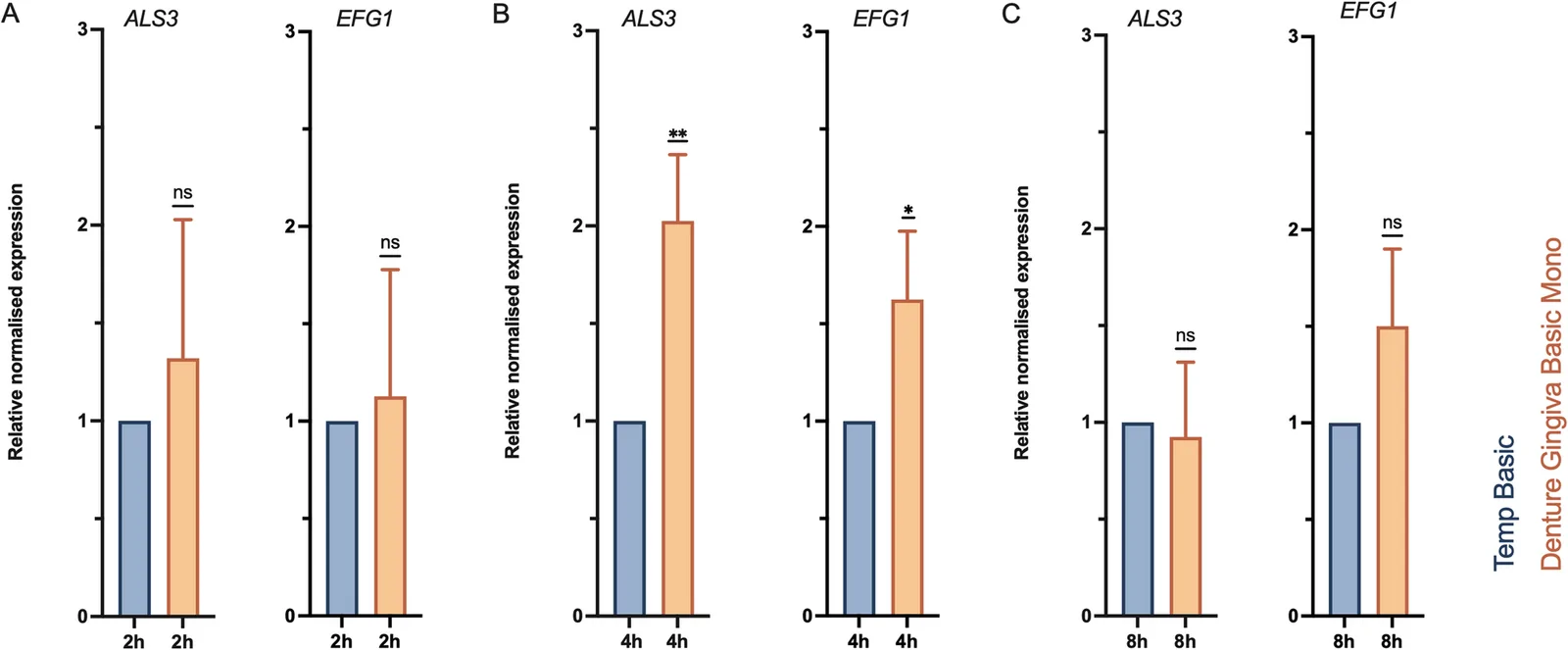
Relative expression of ALS3 and EFG1 in *C. albicans* DSM 1386 on Denture Gingiva Basic Mono compared with Temp Basic. Expression after 2 h (**A**), 4 h (**B**) and 8 h (**C**) was calculated using the ΔΔCq method with *TDH3* as reference gene and Temp Basic set to 1. Statistical analysis was performed using a one-sample t-test against the hypothetical value of 1. Asterisks indicate * *p* < 0.05; ** *p* < 0.01; ns = not significant; *n* = 3

In the reference strain, *ALS3* and *EFG1* expression on Denture Gingiva Basic Mono did not differ significantly from that on Temp Basic after 2 h (p_*ALS3*_ = 0.369; p_*EFG1*_ = 0.686; Fig. 3A). After 4 h, expression of *ALS3* (fold change 2.03 ± 0.34; *p* = 0.009) and *EFG1* (fold change 1.63 ± 0.35; *p* = 0.038) was significantly higher on Denture Gingiva Basic Mono (Fig. 3B). After 8 h, no significant differences were detected (p_*ALS3*_ = 0.724; p_*EFG1*_ = 0.163; Fig. 3C).

**Fig. 4:**
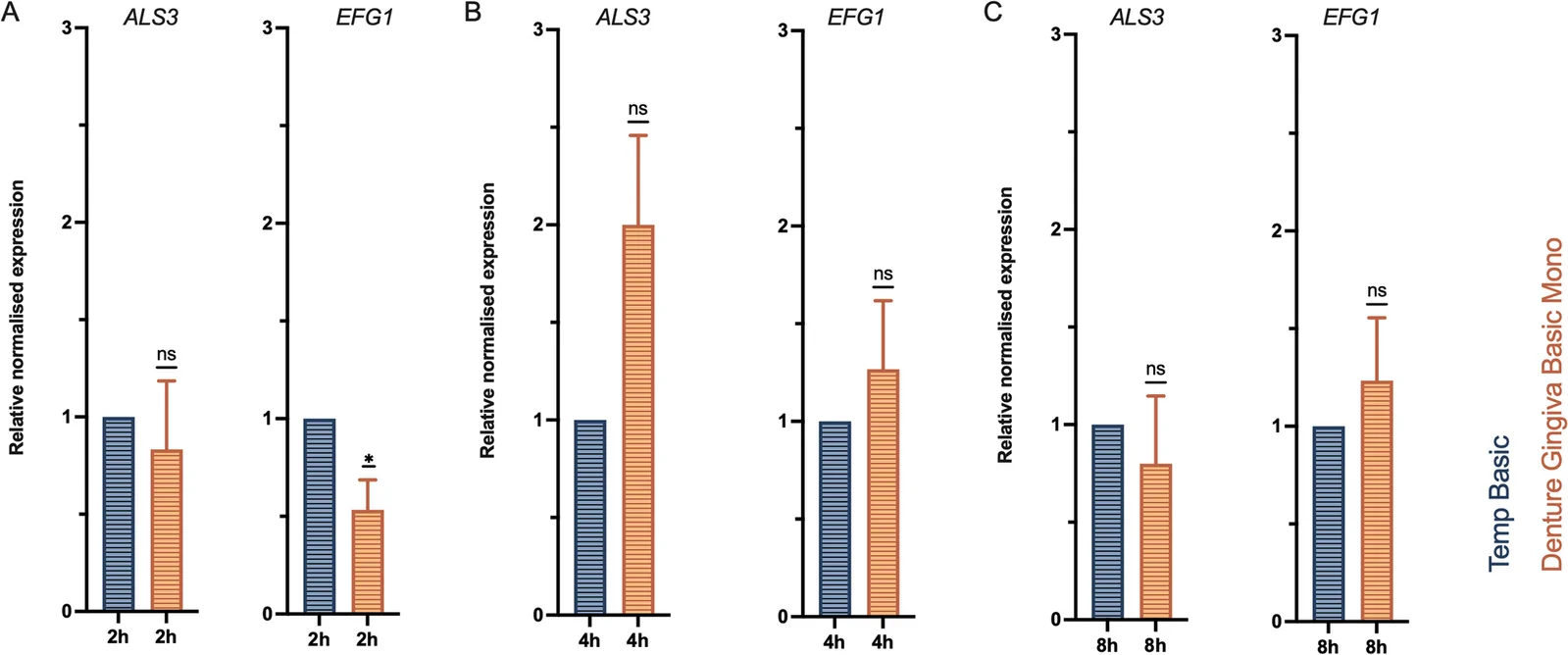
Relative expression of ALS3 and EFG1 in clinical *C. albicans* isolate 1 on Denture Gingiva Basic Mono compared with Temp Basic. Expression after 2 h (**A**), 4 h (**B**) and 8 h (**C**) was calculated using the ΔΔCq method with *TDH3* as reference gene and Temp Basic set to 1. Statistical analysis was performed using a one-sample t-test against the hypothetical value of 1. Asterisks indicate * *p* < 0.05; ns = not significant; *n* = 3

In clinical isolate 1, *ALS3* and *EFG1* expression on Denture Gingiva Basic Mono was likewise higher than on Temp Basic after 4 h, although the differences were not significant (p_*ALS3*_ = 0.063; p_*EFG1*_ = 0.319; Fig. 4B). After 2 h, *EFG1* expression was significantly lower on Denture Gingiva Basic Mono (fold change 0.53 ± 0.15; *p* = 0.034), whereas *ALS3* expression did not differ (*p* = 0.498; Fig. 4A); after 8 h, no significant differences were detected (p_*ALS3*_ = 0.423; p_*EFG1*_ = 0.336; Fig. 4C). Clinical isolate 2, analyzed in a single experiment, also showed higher *ALS3* and *EFG1* expression on Denture Gingiva Basic Mono after 4 h (Fig. S2).

## Discussion

The present study investigated whether denture base materials fabricated by conventional, subtractive and additive manufacturing were associated with differences in the adhesion, biofilm formation and gene expression of *C. albicans*. Initial adhesion was assessed under passive, non-growth-permissive conditions using PBS, which restricts *C. albicans* metabolism and thereby promotes attachment primarily through physicochemical interactions between the fungal cell surface and the substrate material. These interactions are known to be governed by multiple surface parameters, including surface roughness, SFE, surface charge, and nanoscale topographic features [13].

As all materials had R_a_ values below 0.2 μm, a threshold below which roughness is considered to exert negligible influence on microbial adhesion [14], roughness is unlikely to account for the differences observed. SEM analysis showed polishing grooves on all materials, which appeared more pronounced on Denture Base Resin. However, this difference did not appear to substantially affect initial colonization, as Denture Base Resin did not display outlying adhesion values relative to the other materials.

Nevertheless, initial adhesion varied between the materials, suggesting that material-related factors other than roughness contribute to early colonization, consistent with previous reports on the influence of material-specific physicochemical properties on microbial adhesion [13, 38–44].

Previous studies and reviews mostly reported lower *C. albicans* adhesion on milled and higher adhesion on 3D-printed denture base resins, which has been attributed mainly to the layer-wise surface texture of printed materials [30, 31]. In the present study, however, the milled material Temp Basic showed the highest mean adhesion, whereas the printed materials did not differ from most other materials. This is consistent with Vetsch et al., who observed higher biofilm formation on a milled than on a heat-polymerized resin [36]. As all specimens were polished to comparable roughness, the printing-related surface texture was eliminated in our setting. The results therefore do not necessarily contradict previous reports but indicate that, once topography is standardized, material-specific properties such as resin chemistry and SFE govern adhesion and may vary considerably within one manufacturing category [30, 45]. Clinically, however, the tissue surface of dentures is usually not polished, so that manufacturing-related roughness remains relevant [45].

A particularly noteworthy finding of the present study is the observation that Temp Basic, despite exhibiting the lowest SFE among all tested materials, supported the highest degree of initial *C. albicans* adhesion. This result is seemingly paradoxical, as lower SFE is generally associated with reduced microbial adhesion due to diminished thermodynamic driving forces for surface colonization [46]. Furthermore, *C. albicans* has been reported to preferentially adhere to surfaces with higher SFE values [13], which would predict the opposite of what was observed here. However, thermodynamic considerations predict that microorganisms with a low surface free energy, i.e. hydrophobic cells, preferentially adhere to substrates with low SFE [10, 11, 47].

This discrepancy suggests that SFE alone is insufficient to predict early colonization behavior and that additional material-specific factors contribute to the observed differences in adhesion. One potential contributing factor is surface charge, which is known to be influenced by material composition [13, 15, 39, 43, 44, 48] and which was not directly measured in the present study. Given that *C. albicans* cell walls carry a net negative charge under physiological conditions [49, 50], electrostatic interactions with the material surface may modulate adhesion independently of SFE. EDS analysis revealed that all materials predominantly consist of carbon and oxygen, with aluminum additionally detected on four materials, including Temp Basic and Paladon 65. While the aluminum signal on Temp Basic may represent surface contamination rather than a bulk compositional difference, even trace surface elements can alter local surface charge distribution and thereby influence microbial interactions. The elucidation of the precise physicochemical mechanisms underlying the enhanced adhesion on Temp Basic warrants further investigation, including zeta potential measurements and more detailed surface chemical characterization.

Despite the substantially higher initial adhesion observed on Temp Basic, no statistically significant difference in biofilm biomass was detected between Temp Basic and Denture Gingiva Basic Mono after 24 h of incubation, as assessed by crystal violet staining. Thus, for the two materials investigated, higher initial adhesion did not result in greater biofilm biomass after 24 h, suggesting that material-dependent differences in early colonization may be compensated during subsequent stages of biofilm development.

This observation is consistent with previous studies demonstrating that initial adhesion and mature biofilm formation are governed by partially distinct mechanisms and are not always directly correlated [13, 15, 51, 52]. The crystal violet assay quantifies total biofilm biomass, encompassing both cellular and extracellular matrix components, but does not provide information on biofilm architecture, metabolic activity, or transcriptional state. The comparable biomass values observed at 24 h therefore do not preclude the existence of qualitative differences in biofilm composition or maturation between the two materials.

To investigate whether material-dependent differences manifest at the transcriptional level during early biofilm development, gene expression of *ALS3* and *EFG1* was assessed at 2, 4, and 8 h post-incubation. *ALS3* encodes a key adhesin that mediates both cell-to-surface and cell-to-cell interactions during biofilm formation [53, 54], while *EFG1* encodes a central transcriptional regulator of hyphal morphogenesis and biofilm development [55, 56]. No significant differences in the expression of either gene were observed at 2 h, possibly because transcriptional reprogramming requires time following initial surface contact.

At 4 h, however, a marked upregulation of both *ALS3* and *EFG1* was detected in *C. albicans* cells incubated on Denture Gingiva Basic Mono compared to those on Temp Basic, suggesting a stronger induction of genes associated with hyphal morphogenesis and biofilm formation on this material during the early phase of biofilm development. This difference was transient, as expression levels converged by 8 h with no significant differences detected between the two materials.

In both clinical isolates, *ALS3* and *EFG1* expression on Denture Gingiva Basic Mono was likewise higher after 4 h than on Temp Basic, although the difference was not significant in clinical isolate 1, and clinical isolate 2 was analyzed in a single experiment only (Fig. 4; Fig. S2). The increase after 4 h was thus observed in all three strains, suggesting that the material-associated transcriptional response is not restricted to the reference strain.

One possible, albeit speculative, explanation for these observations may be found in the quorum-sensing biology of *C. albicans*. Cell density has been identified as a critical determinant of *C. albicans* morphogenesis and biofilm maturation, mediated in part by farnesol, a quorum-sensing molecule that accumulates at high cell densities and inhibits the yeast-to-hyphae transition [57].

Given the substantially higher number of initially adherent cells on Temp Basic, local farnesol concentrations at the material surface may have reached inhibitory levels more rapidly, thereby suppressing hyphal induction and the associated upregulation of *ALS3* and *EFG1*. Conversely, the lower initial cell density on Denture Gingiva Basic Mono may have maintained sub-inhibitory farnesol concentrations for a longer period, permitting a more robust transcriptional induction of biofilm-associated genes at 4 h. Such a density-dependent mechanism could represent a compensatory pathway by which *C. albicans* adapts its biofilm development program in response to surface-specific colonization levels, ultimately resulting in comparable biofilm biomass at 24 h irrespective of initial adhesion. As neither farnesol concentrations nor quorum-sensing activity were measured in the present study, this hypothesis requires experimental validation.

Several aspects of the present study should nonetheless be considered when interpreting these findings. Both assays were performed in vitro, and initial adhesion in particular was assessed in PBS rather than culture medium. Although this does not reproduce the dynamic oral environment, it was chosen deliberately to emphasize the passive physicochemical component of attachment while minimizing medium-induced, actively regulated interactions such as adhesin expression; pre-formed adhesins carried over from the preculture may, however, still contribute. Surface charge, though a recognized determinant of adhesion, was not measured directly; the principal physicochemical parameters, like roughness, wettability, and surface free energy were nonetheless characterized, so it represents a complementary refinement rather than an unaddressed confounder. Finally, the study was intentionally confined to *C. albicans*, the principal agent of denture stomatitis, and to two representative markers, *ALS3* and *EFG1*, examined during the early phase in which material-dependent differences were most pronounced.

Additionally, in the oral cavity, denture surfaces are rapidly covered by a salivary conditioning film consisting of adsorbed glycoproteins, mucins, proline-rich proteins and enzymes [58]. Its formation renders hydrophobic surfaces more hydrophilic, alters surface properties differentially across materials [59–61], equalizes surface charges between materials [62] and provides receptors for microbial adhesins [63, 64], so that initial adhesion in vivo results from both non-specific physicochemical interactions (van der Waals, electrostatic and hydrophobic forces) and specific molecular interactions [58]. However, the composition and properties of the pellicle depend on the underlying substrate, so that material characteristics are partly transferred through the pellicle [58]. Saliva modulates *C. albicans* biofilm formation [65], microorganisms can co-aggregate in saliva and attach to existing biofilms, and salivary flow, pH, temperature and composition vary between individuals and are altered by denture treatment itself [66]. In vivo, denture biofilms are polymicrobial, with streptococci and other bacteria interacting with *C. albicans* [67–69], and oral surfaces are colonized by site-specific communities [70]. The present results therefore reflect material-dependent effects under standardized, saliva-free conditions and cannot be directly extrapolated to the clinical situation.

Furthermore, specimens were tested without artificial aging. Thermocycling increases surface roughness and reduces hardness, with the most pronounced changes in 3D-printed and the smallest in milled PMMA, and 3D-printed resins show higher water sorption [71, 72]. Material-dependent differences in colonization may therefore change during clinical service.

Initial adhesion and biofilm formation were assessed using a single reference strain, *C. albicans* DSM 1386, originally isolated in 1978 from a patient with bronchomycosis. *C. albicans* isolates display considerable phenotypic and transcriptional heterogeneity in biofilm formation [73], and *ALS3* expression is highly context-dependent [74]. *ALS3* and *EFG1* expression was therefore additionally analyzed in two clinical isolates from the oral cavity, which also showed higher expression on Denture Gingiva Basic Mono after 4 h; however, this difference was not significant in clinical isolate 1, and clinical isolate 2 was analyzed in a single experiment only. Nevertheless, confirmation with further clinical isolates, *ALS3* deletion mutants and in vivo models is required to establish a causal role of *ALS3* regulation in denture stomatitis.

Future studies should investigate these effects under more physiologically relevant conditions, including salivary conditioning films, polymicrobial biofilm models, artificially aged specimens, further clinical isolates and extended time course analyses, to better understand the clinical relevance of material-dependent differences in *C. albicans* colonization behavior.

## Conclusions

Within the limitations of this in vitro study, the denture base materials investigated were associated with differences in *C. albicans* colonization behavior, although surface roughness was standardized. Initial adhesion varied between materials and was highest on the milled material Temp Basic, which also showed the lowest SFE; SFE alone therefore did not explain initial adhesion. For Temp Basic and Denture Gingiva Basic Mono, the materials with the highest and lowest initial adhesion, these differences did not translate into differences in biofilm biomass after 24 h. On Denture Gingiva Basic Mono, *ALS3* and *EFG1* expression was higher after 4 h, significantly in the reference strain and in the same direction in two clinical isolates. Evaluating microbial behavior beyond initial adhesion, including gene expression, may therefore help to characterize material-microbe interactions. As saliva, polymicrobial biofilms and material aging were not considered, the clinical relevance of these findings remains to be confirmed.

## Supplementary Information

Supplementary Material 1.

## Data Availability

The datasets generated and analyzed during the current study are available from the corresponding author on reasonable request.

## Abbreviations

ALS3: Agglutinin-like sequence 3
ANOVA: Analysis of variance
C. albicans: Candida albicans
cDNA: Complementary deoxyribonucleic acid
DNase: Deoxyribonuclease
DSMZ: German Collection of Microorganisms and Cell Cultures
EDS: Energy-dispersive X-ray spectroscopy
EFG1: Enhanced filamentous growth 1
OD: Optical density
PBS: Phosphate-buffered saline
PFA: Paraformaldehyde
qPCR: Quantitative real-time polymerase chain reaction
Ra: Arithmetic mean surface roughness
RNA: Ribonucleic acid
RT-qPCR: Reverse transcription quantitative polymerase chain reaction
SEM: Scanning electron microscopy
SFE: Surface free energy
TDH3: Triose-phosphate dehydrogenase 3
YM: Yeast medium
