# Supplementary material for: Candida albicans adhesion, biofilm formation and gene expression on conventional, milled and 3D-printed denture base materials: an in vitro study
Source: BMC Oral Health. 2026 Nov 4;26:2190. doi: 10.1186/s12903-026-10345-y (PMC13733116; doi:10.1186/s12903-026-10345-y)
Supplement: Supplementary file 1 [file 12903_2026_10345_MOESM1_ESM.docx]

**Supplementary Information**

**Table S1** Overview of material-specific polishing protocols

| Denture base material | Rotational speed [rpm] | Force [N] | Time [s] | Grit | Number of polishing cycles |
| --- | --- | --- | --- | --- | --- |
| Paladon 65 | 50 | 5 | 45 | 400 | 2 |
|  | 50 | 5 | 60 | 800 | 16 |
|  | 50 | 5 | 360 | 1200 | 4 |
|  | 50 | 5 | 360 | 4000 | 4 |
| PalaXpress | 50 | 5 | 45 | 400 | 2 |
|  | 50 | 5 | 60 | 800 | 16 |
|  | 50 | 5 | 360 | 1200 | 4 |
|  | 50 | 5 | 360 | 4000 | 4 |
| V-Print dentbase | 50 | 5 | 45 | 400 | 2 |
|  | 50 | 5 | 45 | 800 | 2 |
|  | 50 | 5 | 180 | 1200 | 2 |
|  | 50 | 5 | 240 | 4000 | 2 |
|  | 150 | 5 | 360 | 1200 | 4 |
|  | 150 | 5 | 360 | 4000 | 14 |
| Denture Base Resin | 50 | 5 | 360 | 400 | 1 |
|  | 50 | 5 | 360 | 800 | 1 |
|  | 50 | 5 | 360 | 1200 | 2 |
|  | 50 | 5 | 120 | 4000 | 4 |
|  | 150 | 5 | 360 | 1200 | 2 |
|  | 150 | 5 | 360 | 4000 | 12 |
| Temp Basic | 50 | 5 | 45 | 400 | 2 |
|  | 50 | 5 | 60 | 800 | 16 |
|  | 50 | 5 | 360 | 1200 | 4 |
|  | 50 | 5 | 360 | 4000 | 4 |
| Denture Gingiva Basic Mono | 50 | 5 | 45 | 400 | 2 |
|  | 50 | 5 | 60 | 800 | 16 |
|  | 50 | 5 | 360 | 1200 | 4 |
|  | 50 | 5 | 360 | 4000 | 4 |
| M-PM Disc | 50 | 5 | 45 | 400 | 2 |
|  | 50 | 5 | 60 | 800 | 16 |
|  | 50 | 5 | 360 | 1200 | 4 |
|  | 50 | 5 | 360 | 4000 | 4 |
| Ivotion Base | 50 | 5 | 45 | 400 | 2 |
|  | 50 | 5 | 60 | 800 | 16 |
|  | 50 | 5 | 360 | 1200 | 4 |
|  | 50 | 5 | 360 | 4000 | 4 |

*Note.* The following grinding papers were used: DEMA-PL grinding paper discs adhesive (Metkon Instruments Inc., Bursa, Turkey), WS Flex 18 C SK grit 400 and 1200 as well as WS Flex 18 CMS grit 4000 (Hermes Schleifmittel GmbH, Hamburg, Germany).

**Table S2** Elemental surface composition of the denture base materials determined by EDS

|  | PalaXpress | | Paladon 65 | | V-Print dentbase | | Denture Base Resin | | Temp Basic | | Denture Gingiva Basic Mono | | M-PM Disc | | Ivotion Base | |
| --- | --- | --- | --- | --- | --- | --- | --- | --- | --- | --- | --- | --- | --- | --- | --- | --- |
| Element | **wt** [%] | **at** [%] | **wt** [%] | **at** [%] | **wt** [%] | **at** [%] | **wt** [%] | **at** [%] | **wt** [%] | **at** [%] | **wt** [%] | **at** [%] | **wt** [%] | **at** [%] | **wt** [%] | **at** [%] |
| C | 26.87 | 33.09 | 26.58 | 33.00 | 13.97 | 26.58 | 13.72 | 18.35 | 26.78 | 33.03 | 27.01 | 33.17 | 26.84 | 33.09 | 26.96 | 33.13 |
| O | 71.60 | 66.17 | 71.06 | 65.99 | 37.41 | 53.16 | 51.52 | 60.53 | 71.34 | 66.06 | 71.96 | 66.34 | 71.50 | 66.10 | 71.84 | 66.26 |
| Al | 0 | 0 | 1.30 | 0 | 1.46 | 1.30 | 8.21 | 7.58 | 1.06 | 0 | 0 | 0 | 0 | 0 | 0 | 0 |
| Si | 0 | 0 | 0 | 0 | 19.35 | 16.83 | 0 | 0 | 0 | 0 | 0 | 0 | 0 | 0 | 0 | 0 |
| K | 0 | 0 | 0 | 0 | 0 | 0 | 1.5 | 1.10 | 0 | 0 | 0 | 0 | 0 | 0 | 0 | 0 |
| Ti | 0 | 0 | 0 | 0 | 0 | 0 | 10.23 | 5.11 | 0 | 0 | 0 | 0 | 0 | 0 | 0 | 0 |
| Fe | 0 | 0 | 0 | 0 | 0 | 0 | 5.08 | 2.08 | 0 | 0 | 0 | 0 | 0 | 0 | 0 | 0 |
| Mn | 0 | 0 | 0 | 0 | 0 | 0 | 3.65 | 1.70 | 0 | 0 | 0 | 0 | 0 | 0 | 0 | 0 |
| P | 0 | 0 | 0 | 0 | 0 | 0 | 5.05 | 3.06 | 0 | 0 | 0 | 0 | 0 | 0 | 0 | 0 |

*Note.* Inclusion threshold: ≥ 1, expressed in weight percent (wt%) and atomic percent (at%)

**Table S3** Surface roughness, surface free energy and initial adhesion of C. albicans (mean ± standard deviation)

| **Denture base material** | **R_a_ [µm]** | **SFE [mN/m]** | **Initial adhesion [cells/0.1 mm²]** |
| --- | --- | --- | --- |
| PalaXpress | 0.047 ± 0.017 | 61.28 ± 1.54 | 282.86 ± 56.21 |
| Paladon 65 | 0.034 ± 0.007 | 61.89 ± 0.67 | 317.03 ± 114.33 |
| V-Print dentbase | 0.041 ± 0.007 | 60.44 ± 2.67 | 293.84 ± 94.00 |
| Denture Base Resin | 0.037 ± 0.002 | 63.12 ± 1.77 | 374.66 ± 135.25 |
| Temp Basic | 0.043 ± 0.010 | 52.58 ± 1.97 | 466.54 ± 110.49 |
| Denture Gingiva Basic Mono | 0.035 ± 0.016 | 63.84 ± 1.04 | 276.43 ± 114.24 |
| M-PM Disc | 0.049 ± 0.006 | 63.56 ± 1.26 | 394.94 ± 148.89 |
| Ivotion Base | 0.055 ± 0.009 | 61.37 ± 0.12 | 356.07 ± 135.55 |

*Note.* R_a_: n = 5 specimens (five measuring points each); SFE: n = 5 specimens; initial adhesion: n = 11.

**Table S4** Biofilm formation and relative ALS3 and EFG1 expression on Temp Basic and Denture Gingiva Basic Mono (mean ± standard deviation)

| **Parameter** | **Strain** | **Time** | **Temp Basic** | **Denture Gingiva Basic Mono** |
| --- | --- | --- | --- | --- |
| Biofilm biomass (relative) | DSM 1386 | 24 h | 1.74 ± 1.58 | 1 |
| *ALS3* (fold change) | DSM 1386 | 2 h | 1 | 1.32 ± 0.71 |
| *ALS3* (fold change) | DSM 1386 | 4 h | 1 | 2.03 ± 0.34 |
| *ALS3* (fold change) | DSM 1386 | 8 h | 1 | 0.93 ± 0.39 |
| *ALS3* (fold change) | Clinical isolate 1 | 2 h | 1 | 0.83 ± 0.35 |
| *ALS3* (fold change) | Clinical isolate 1 | 4 h | 1 | 2.00 ± 0.46 |
| *ALS3* (fold change) | Clinical isolate 1 | 8 h | 1 | 0.80 ± 0.35 |
| *ALS3* (fold change) | Clinical isolate 2 | 2 h | 1 | 0.70 |
| *ALS3* (fold change) | Clinical isolate 2 | 4 h | 1 | 1.90 |
| *ALS3* (fold change) | Clinical isolate 2 | 8 h | 1 | 1.30 |
| *EFG1* (fold change) | DSM 1386 | 2 h | 1 | 1.13 ± 0.65 |
| *EFG1* (fold change) | DSM 1386 | 4 h | 1 | 1.63 ± 0.35 |
| *EFG1* (fold change) | DSM 1386 | 8 h | 1 | 1.50 ± 0.40 |
| *EFG1* (fold change) | Clinical isolate 1 | 2 h | 1 | 0.53 ± 0.15 |
| *EFG1* (fold change) | Clinical isolate 1 | 4 h | 1 | 1.27 ± 0.35 |
| *EFG1* (fold change) | Clinical isolate 1 | 8 h | 1 | 1.23 ± 0.32 |
| *EFG1* (fold change) | Clinical isolate 2 | 2 h | 1 | 0.30 |
| *EFG1* (fold change) | Clinical isolate 2 | 4 h | 1 | 2.30 |
| *EFG1* (fold change) | Clinical isolate 2 | 8 h | 1 | 1.40 |

*Note.* Fold changes were calculated using the ΔΔCq method with *TDH3* as reference gene and Temp Basic set to 1. Biofilm biomass was normalized to Denture Gingiva Basic Mono (set to 1); n = 7; gene expression: n = 3 biological replicates for DSM 1386 and clinical isolate 1; clinical isolate 2 was analyzed in a single experiment (n = 1, individual values).

**Figure S1:** **Association between SFE and biofilm biomass.**

Scatter plot of SFE and relative biofilm biomass after 24 h of incubation with *C. albicans* on Temp Basic and Denture Gingiva Basic Mono; lines indicate the linear regression for each material. Neither Spearman’s correlation nor multiple linear regression showed a significant association. n = 20.

**Figure S2: Relative expression of *ALS3* and *EFG1* in clinical *C. albicans* isolate 2 on Denture Gingiva Basic Mono compared with Temp Basic.**

Expression after 2 h (A), 4 h (B) and 8 h (C) was calculated using the ΔΔCq method with *TDH3* as reference gene and Temp Basic set to 1. Single experiment (n = 1); no statistical analysis was performed.
